# Prevalence of hemoglobin S trait among blood donors: a cross-sectional study

**DOI:** 10.1186/s13104-015-1583-0

**Published:** 2015-10-19

**Authors:** Samuel Antwi-Baffour, Ransford Owiredu Asare, Jonathan Kofi Adjei, Ransford Kyeremeh, David Nana Adjei

**Affiliations:** Department of Medical Laboratory Sciences, School of Biomedical and Allied Health Sciences, College of Health Sciences, University of Ghana, P. O. Box KB 143, Korle-Bu, Accra, Ghana

**Keywords:** Hemoglobin, Blood donor, Sickle cell disease, Electrophoresis, Genotype

## Abstract

**Background:**

Sickle cell trait (SCT) or Hemoglobin S (HbS) trait which is due to inheritance of an abnormal hemoglobin (Hb) gene from one parent and a normal gene from the other has been known to be common among people of African descent. Individuals with SCT may find themselves in the blood donor population without knowing their ‘carrier’ status and this may have severe consequences on their health as well as that of a recipient, particularly if they happen to be a sickle cell disease patient. The aim of the study was to determine the prevalence of HbS trait among blood donors.

**Results:**

This cross-sectional study employed convenience sampling method to recruit subjects. A total of 150 prospective and healthy blood donors comprising 138 males and 12 females were involved in the study. Two (2) ml of venous blood was collected from each donor into K_3_EDTA tubes and analyzed using the sodium metabisulphite slide test and cellulose acetate Hb electrophoresis at alkaline pH (8.6) for Hb genotypes. Statistical Package for Social Sciences version 20.0 (SPSS 20.0) and Chi square were used to analyse the data obtained. Out of the 150 blood donors, 133 (88.7 %) tested negative for sickling (131 were genotype AA and 2 were AC) and 17 (11.3 %) tested positive for sickling, all of whom were genotype AS.

**Conclusion:**

The results of the study showed the existence of SCT among the blood donor population sampled. Taking blood from such people can harm their health as well as that of the recipient if they happen to be sickle cell disease (SCD) patients. It is therefore recommended that blood donors as well as donated blood units should be screened for SCT to avoid causing any harm to both the donor and recipient.

## Hypothesis

Sickle cell disease (SCD) refers to a group of disorders that affects hemoglobin (Hb), causing them to form abnormal hemoglobin S (HbS) molecules [1]. Affected people inherited the abnormal gene (HbS) from both parents. A person who inherits an abnormal gene from one parent and a normal gene from the other has a sickle cell trait (SCT) or is said to be a carrier of SCD [2]. The gene for sickle cell disease is more common in Sub-Sahara Africa, Mediterranean countries, the Middle East and India [3]. The Hispanic population of both Spanish or Latin American descent is also at risk of the disease—at least 1 in 180 Hispanic births have sickle cell trait [4], [5].

Reports from studies conducted in Ghana show that about 25–30 % of the Ghanaian population carry the sickle cell trait and 2 % of newborns have sickle cell disease [6, 7]. Sickle cell disease is a major public health concern, having socio-economic implications for the affected child as well as their family. Patients with SCD are often hospitalised for long periods [6, 8]. Also, death in early childhood as a result of SCD is high in Africa partly due to lack of comprehensive healthcare [9, 10].

Blood transfusion is an important act that saves millions of lives each year [11]. It is estimated that over 90 million blood units are collected worldwide each year [12]. According to the WHO, four main types of blood donors can be identified. They include: voluntary non-remunerated donors; family/replacement/directed donors; paid/commercial donors and autologous donors [13]. In order to ensure a safe and sufficient blood supply, the WHO adopted a strategy that aimed to implement a national policy for each African country by 2012 [14]. This included the recruitment of regular voluntary, healthy and non-remunerated blood donors. However, with the high prevalence (25–30 %) of sickle cell trait in Ghana, people who have the sickle cell trait may enrol as prospective blood donors since majority of them are asymptomatic and in stable condition [15, 16]. They may therefore find themselves in the donor population unknowingly. This may have severe consequences on their health after they have donated blood as well as that of the recipients particularly if they happen to have sickle cell disease.

Patients with SCD may require frequent blood transfusion to treat complications associated with the disease [17, 18]. However, transfusing blood containing HbS to a SCD patient can increase the proportion of sickled red cells in the person’s circulation, inducing further sickling and causing occlusions in the microcirculation [19]. This deprives the tissues and organs of oxygen, resulting in vaso-occlusive crisis and affecting proper management of the patient [20]. In view of the above, this study sought to determine the prevalence of HbS trait among blood donors to help in ensuring efficient and safe blood donation and transfusion.

## Methods

### Study design

The study was a cross-sectional study carried out from May to July, 2014.

### Study setting/participants

The study subjects were apparently healthy individuals between the ages of 17 and 60 years who came to donate blood at the southern area blood centre (SABC) of the Korle-bu Teaching Hospital, Accra. They included voluntary and replacement blood donors.

### Ethical consideration

Ethical clearance for this research was sought from the Ethics and Protocol Review Committee at the School of Biomedical and Allied Health Sciences, University of Ghana, Legon. All the participants gave their informed consent before their samples were collected.

### Materials

Some of the materials needed for the work include: EDTA tubes, 2 % Sodium metabisulphite, Physiological saline (0.85 %), Electrophoretic tank and power pack (Consort EV 243), Centrifuge, Cellulose acetate paper/membrane, Tris–EDTA Borate (TEB) buffer (pH 8.6), Carbon tetrachloride (CCl_4_), 5 % Acetic acid, Ponceau S stain.

### Sample collection/analysis

One hundred and fifty (150) subjects, involving 138 males and 12 females were recruited using convenience sampling method. Two millilitres (2 ml) of blood was collected from each of the participants during blood donation into labelled tri-potassium ethylene diamine tetra-acetic acid (K_3_EDTA) tubes and mixed gently. All samples were kept at 4–8 °C after which they were transported to the laboratory for analysis. Samples were processed within 24 h after collection.

### Sickling test (Sodium metabisulphite method)

Equal volumes of well-mixed EDTA anticoagulated blood and 2 % sodium metabisulphite were mixed on a cleaned labelled slide, covered with a cover slip and incubated at room temperature for at least 30 min. It was then examined microscopically, using the 40× objective for sickle cells. Sickle cells appeared crescent-shaped [21]. Positive and negative controls, from a known sickle cell trait person and a person without the sickle cell trait respectively, were set-up alongside the test.

### Hemoglobin electrophoresis (cellulose acetate method at alkaline pH)

The blood samples were centrifuged at 1500*g* for 5 min and the plasma removed. The red cells were then washed four times with physiological saline (0.85 %). Few drops of distilled water were added to lyse the cells after which four drops of CCl_4_ was added, vortexed and centrifuged at 1500*g* for 20 min to separate the hemoglobin. The Hemolysate was transferred into a clean glass test tube [21].

Each compartment of the electrophoretic tank was filled with Tris–EDTA Borate (TEB) buffer (pH 8.6) to a depth of about 2.5 cm. The cellulose acetate membrane was impregnated with TEB buffer and blotted with a tissue paper to remove excess buffer, but not allowed to dry. By means of an applicator, the control and test hemolysate samples were applied on the cellulose acetate membrane and carefully introduced onto the frame of the electrophoretic tank, with both ends in contact with the buffer. The lid of the tank was replaced and the tank connected to a power supply of voltage 250 V and current 50 mA, and allowed to run for 30 min. The power was disconnected and the membrane removed and stained with Ponceau S for 5 min. The excess stain was removed with 3 changes of 3 % acetic acid for 5 min each. The membrane was blotted and allowed to air-dry after which it was labelled and the results read against the controls [22]. A combination of hemolysate from a sickle cell trait (AS) and HbC trait samples (ASC) served as the control.

### Data analysis

Data collected was entered into Microsoft Office Excel 2010 and analysed using Statistical Package for Social Sciences version 20.0 (SPSS 20.0). A summary was presented using descriptive statistics such as frequencies, percentages, mean and standard deviations. Chi square analysis was used to establish association between sickling status, Hb genotypes and gender. A *p* value of <0.05 was considered statistically significant.

## Results

A total number of 1300 prospective and mainly replacement blood donors presented to donate blood during the period of this study. Out of these, 1066 (82 %) were males and 234 (18 %) females. The study examined one hundred and fifty (150) of these donors who consented to take part in the study. Out of these, 138 (92 %) were males and 12 (8 %) females respectively. The mean age of the participants was 30.5 ± 8.4 years. The youngest participant was 18 years and the oldest was 51 years. The age group of 20–24 was the highest representing 28.7 % of the total number of participants (Table 1). Only four (4) participants (first time donors) representing 2.7 % knew their sickling status and Hb genotype while the remaining 146 (97.3 %) did not know both their sickling status and Hb genotypes. Ninety-six (64 %) of them had not donated blood before but the remaining 54 (36 %) had (Table 1). Out of the 54 that have had previous blood donation experience, 29 had donated blood once, 14 twice, 7 thrice and 4 four times. In terms of types of donors, Sixteen (16) participants representing 10.7 % were voluntary donors and the remaining 134 representing 89.3 % were replacement donors. There were no commercial and autologous donors.

**Table 1:** Distribution of demographic characteristics of participants

Variables	Numbers	Percentage (%)
Gender		
Male	138	92.0
Female	12	8.0
Total	150	100
Age group		
15–19	3	2.0
20–24	43	28.7
25–29	37	24.7
30–34	19	12.7
35–39	19	12.7
40–44	17	11.3
≥45	12	8
Total	150	100
Knowledge of sickling/Hb genotype status		
Yes	4	2.7
No	146	97.3
Total	150	100
Previous donations		
Yes	54	36.0
No	96	64.0
Total	150	100

**Fig. 1:**
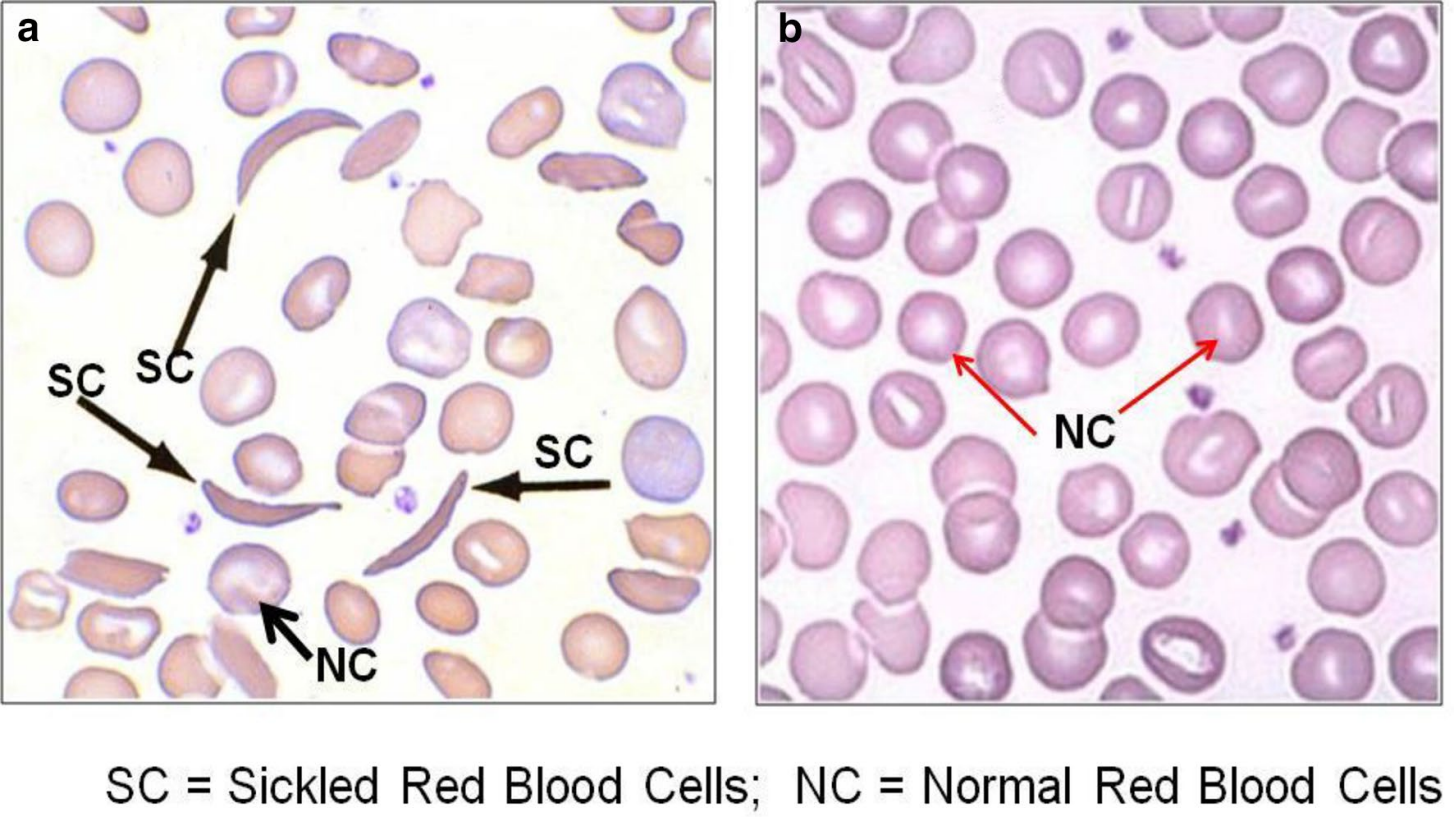
A figure of photomicrograph results for blood obtained from a person without and with sickle cell trait. **a** Shows sickled red blood cells (*black arrowed*) from a person with sickle cell trait under low oxygen tension. **b** Represent normal red blood cells (*red arrowed*) from a person with no sickle cells trait

**Fig. 2:**
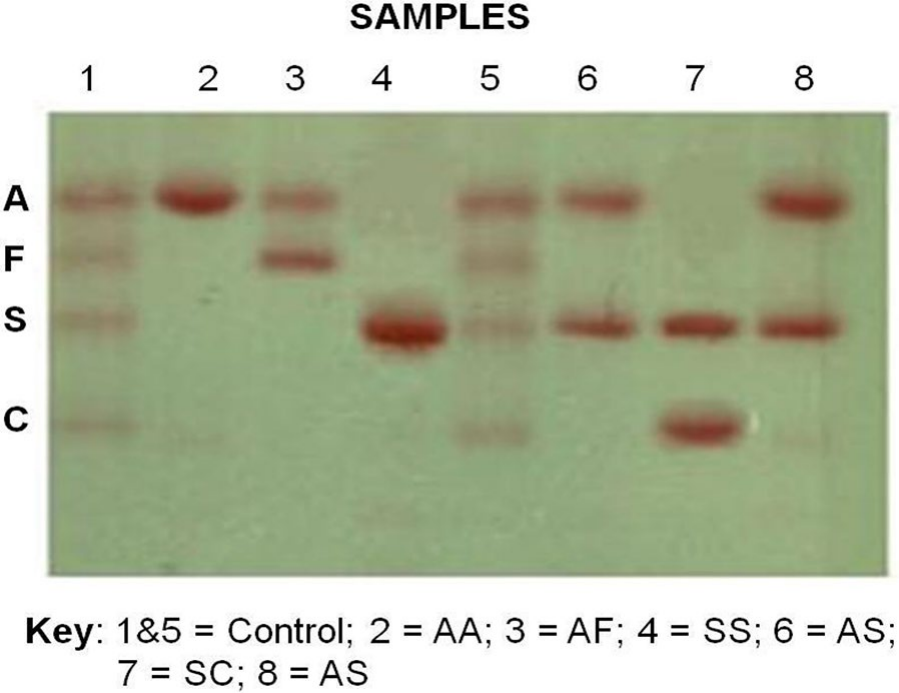
A figure of cellulose acetate Hb electrophoresis from a person without and others with sickle cell trait and disease. The figure represents 6 sickling positive samples (*2*, *3*, *4*, *6*, *7* and *8*) and control samples (*1* and *5*). The control samples were for genotypes AFSC whilst sample *2* was for genotype AA, *3* for AF, *4* for SS, *6* for AS, *7* for SC and *8* for AS

For the sickling status of the participants, seventeen (17) representing 11.3 % tested positive for sickling (Fig. 1a) while the remaining 133 (88.7 %) were sickling negative (Fig. 1b) as shown in Table 2. Twelve (8.7 %) of the 138 male participants tested positive for sickling with the remaining 126 (91.3 %) testing negative. Also, out of 12 females, 5 (41.7 %) tested positive for sickling and 7 (58.3 %) were sickling negative as shown in Table 2 below. Out of the total 150 donors, 131 (87.3 %), 2 (1.3 %) and 17 (11.3 %) were AA, AC and AS respectively. Subsequently, 124 (89.9 %) of the males were AA, 2 (1.4 %) were AC and 12 (8.7 %) were AS, then 7 (58.3 %) of the females were AA and 5 (41.7 %) were AS with no AC (Fig. 2) (Table 2).

**Table 2:** Gender distribution of sickling status and hemoglobin genotype of blood donors

Gender	Sickling status		Hemoglobin genotype			Total
	Negative	Positive	AA	AC	AS	
Male	126 (91.3 %)	12 (8.7 %)	124 (89.9 %)	2 (1.4 %)	12 (8.7 %)	138 (100 %)
Female	7 (58.3 %)	5 (41.7 %)	7 (58.3 %)	0 (0.0 %)	5 (41.7 %)	12 (100 %)
Total	133 (88.7 %)	17 (11.3 %)	131 (87.3 %)	2 (1.3 %)	17 (11.3 %)

## Discussion and conclusion

Blood transfusion is a therapeutic procedure but can be harmful instead of saving lives. Every blood donation and transfusion carries a potential risk for both the donor and recipient [23, 24]. Individuals with certain medical defects may unknowingly find themselves in the donor population which may have effect on their health as well as that of the recipient [19]. The Blood Transfusion Services therefore, have a duty of care towards blood donors and recipients to make sure any contraindications are avoided.

Out of the total 150 blood donors involved in this study, 138 (92 %) were males and 12 (8 %) were females giving a male to female ratio of 12:1. This was high compared to the gender distribution in another study that had a male to female ratio of 4:1 from a population of 314 [16, 25]. The greater proportion of male donors compared to females in our study may be attributed to the fact that females are not very much encouraged to participate in blood donations, be it voluntary or replacement due to certain socio-cultural beliefs. Other physiological conditions may also exclude females from blood donation including pregnancy, lactation and menstruation [25]. Age distribution also showed that, the age group of 20–24 years had the highest number of blood donors (28.7 %) which is similar to the findings in a study by Omisakin et al. that had age group of 15–24 years (53.8 %) with the highest number of donors [16].

Only 4 (2.7 %) of the participants had knowledge of their sickle cell status and Hb genotype, which agrees with a study by Lippi et al. that stated that most blood donors, especially those with SCT are not aware of their sickle cell status [26]. This is because individuals with SCT are usually asymptomatic with most of their hematological parameters such as hemoglobin and red blood cell indices within the normal range [27]. Those with the knowledge of their sickling status were first time donors and presented themselves for donation because they were not aware their sickling status had any bearing on them as blood donors. The prevalence of SCT—(AS) of 11.3 % in this study was found to be lower than the 25–30 % quoted for Ghana and 20–40 % for Africa in general [7, 15]. Also, considered low in relation to studies done by Omisakin et al. and Zaccheaus et al. which had prevalence of HbS trait as 26.1 and 19.68 % respectively [16, 28].

It is recommended that, to achieve a safe and sufficient blood supply, blood should be collected from voluntary non-remunerated donors who have a lower risk of Transfusion-transmissible infections (TTIs) compared to replacement and commercial donors [14]. However, in this study, only 10.7 % were voluntary donors compared to 89.3 % of replacement donors. This falls short of the 80–100 % voluntary donations encouraged in a publication by the centre for disease control of the WHO [29].

There is significant number of people carrying sickle cell trait, especially in Africa and Ghana in particular, who might not be aware of their carrier status before enrolling to be blood donors. It is therefore reasonable to consider the possibility of implementing a practice of routine screening for sickle cell trait in blood donors prior to donating blood or donated blood units. This way transfusion of HbS containing blood to recipients, which can induce further sickling in sickle cell patients, may be avoided. This will go a long way to help in the proper management of sickle cell disease patients and to establish a useful diagnosis of SCT in blood donors.

## Abbreviations

CCl_4_: carbon tetrachloride
EDTA: ethylene diamine tetra-acetic acid
Hb: hemoglobin
HbA: hemoglobin A gene
HbAS: hemoglobin AS (SCT)
HbC: hemoglobin C gene
HbS: hemoglobin S gene
pH: hydrogen ion concentration
SCD: sickle cell disease
SCT: sickle cell trait
SPSS: statistical package for social sciences
TEB: tris-EDTA borate buffer
TTIs: transfusion-transmissible infections
WHO: World Health Organization
